# Sirtuin Inhibitor Cambinol Induces Cell Differentiation and Differently Interferes with SIRT1 and 2 at the Substrate Binding Site

**DOI:** 10.3390/biomedicines11061624

**Published:** 2023-06-02

**Authors:** Deborah Giordano, Bernardina Scafuri, Luigi De Masi, Lucia Capasso, Viviana Maresca, Lucia Altucci, Angela Nebbioso, Angelo Facchiano, Paola Bontempo

**Affiliations:** 1 National Research Council (CNR), Institute of Food Science (ISA), Via Roma 64, 83100 Avellino, Italy; 2 Department of Chemistry and Biology “A. Zambelli”, University of Salerno, Via Giovanni Paolo II 132, 84084 Fisciano, Italy; 3 National Research Council (CNR), Institute of Biosciences and Bioresources (IBBR), Via Università 133, 80055 Portici, Italy; 4 Department of Precision Medicine, University of Campania “Luigi Vanvitelli”, Via L. De Crecchio 7, 80138 Naples, Italypaola.bontempo@unicampania.it (P.B.)

**Keywords:** cell differentiation, epigenetics, post-translational modification, acetylation, HDAC inhibitor, molecular simulation, protein–ligand docking, active site pocket, binding affinity

## Abstract

Epigenetic mechanisms finely regulate gene expression and represent potential therapeutic targets. Cambinol is a synthetic heterocyclic compound that inhibits class III histone deacetylases known as sirtuins (SIRTs). The acetylating action that results could be crucial in modulating cellular functions via epigenetic regulations. The main aim of this research was to investigate the effects of cambinol, and its underlying mechanisms, on cell differentiation by combining wet experiments with bioinformatics analyses and molecular docking simulations. Our in vitro study evidenced the ability of cambinol to induce the differentiation in MCF-7, NB4, and 3T3-L1 cell lines. Interestingly, focusing on the latter that accumulated cytoplasmic lipid droplets, the first promising results related to the action mechanisms of cambinol have shown the induction of cell cycle-related proteins (such as p16 and p27) and modulation of the expression of Rb protein and nuclear receptors related to cell differentiation. Moreover, we explored the inhibitory mechanism of cambinol on human SIRT1 and 2 performing in silico molecular simulations by protein–ligand docking. Cambinol, unlike from other sirtuin inhibitors, is able to better interact with the substrate binding site of SIRT1 than with the inhibition site. Additionally, for SIRT2, cambinol partially interacts with the substrate binding site, although the inhibition site is preferred. Overall, our findings suggest that cambinol might contribute to the development of an alternative to the existing epigenetic therapies that modulate SIRTs.

## 1. Introduction

The interest in the involvement of epigenetic alterations on the development of metabolic and oncological diseases is constantly growing [1]. Epigenetic phenomena regulate genes through chemical processes that do not involve changes in the DNA sequence, but can strongly modify the phenotype of the individual and its progeny [2,3]. Covalent modifications of histone core, such as acetylation on ε-N-amino groups of lysine residues in the N-terminal tails, are key mechanisms that reversibly allow the alteration of the physical accessibility of macromolecular complexes to the genome by being responsible for the regulation of the degree of gene functioning. In this way, “closed” chromatin (heterochromatin) mediates transcriptional repression, while “open” chromatin (euchromatin) includes transcriptionally active genes. The acetylation levels of the histone core are controlled through the balance of two counteracting enzyme activities: histone acetyltransferase (HAT) and histone deacetylase (HDAC) [3]. Four classes of HDAC have been identified in eukaryotes according to their structural homology. More specifically in mammals, class III HDACs have a catalytic activity that is dependent on nicotinamide adenine dinucleotide (NAD^+^) and are better known as Sir2 proteins or sirtuins (SIRTs) to indicate that their protein family is homologous to the yeast protein Sir2 (Silent Information Regulator 2) involved in gene silencing, chromosomal stability, and ageing [4]. In the human genome, there are seven different homologous genes of NAD^+^-dependent deacetylases: SIRT1–SIRT7. These share a catalytic region of about 270 amino acids formed by a Zn-binding domain and a Rossmann fold hosting the NAD^+^ cofactor. SIRTs can combine deacetylation with the hydrolysis of NAD^+^ by forming 2′-O-acetyl-ADP-ribose and releasing nicotinamide (NAM), a feedback inhibitor of SIRTs. These enzymes exert their deacetylase activity on a variety of substrates: transcription factors, tumor suppressors (p53), and regulators of metabolism. It has been observed that SIRTs promote cell survival during stress through the deacetylation of histones and non-histone proteins that are essential to cell cycle regulation.

In mammals, SIRTs are also involved in cell differentiation and adipogenesis through interactions with the peroxisome proliferator-activated receptor-γ (PPARγ), a member of a class of nuclear hormone receptors regulating adipogenesis [5]. The function of SIRT1 in adipocytes also involves the repression of PPARγ through the interaction with the cofactors NCoR (nuclear receptor co-repressor) and SMRT (silencing mediator of retinoid and thyroid hormone receptors), which leads to the repression of adipogenesis, the reduction in lipolysis, and the mobilization of fatty acids [5]. The activity of SIRT2 is involved in the regulation of metabolism: the lowering of the levels of this protein in adipocytes causes an overexpression of C/EPBα and PPARγ and promotes adipogenesis [6]. SIRT3 is involved in the metabolism of carbohydrates, amino acids, fats, and in the tricarboxylic acid cycle for its deacetylase activity [7]. Each of these SIRT isoforms carries out its own function located to a different cell compartment, so SIRT1 is mostly allocated in the nucleus, SIRT2 in the cytoplasm, and SIRT3 in the mitochondria. In addition, as more recently and interestingly shown, different SIRTs prefer to cleave specific lysine acylations on ε-N-amino groups [8,9].

The role and functions of SIRTs in cancer development have not yet been fully elucidated, above all because they play different roles in different kinds of cancer. It is known that SIRT1 has an inhibitory effect on the p53 tumor suppressor and on other genes involved in the stress response, suggesting a pro-carcinogenic role of this SIRT. Furthermore, antisense oligonucleotides directed against SIRT1 induce apoptosis in lung cells, showing its therapeutic potential in lung cancer [10]. SIRT2 could also represent a possible target for anticancer drugs, given its ability to modulate the cell cycle. The fact that the levels of this enzyme are drastically decreased in human glioma cells suggests a function comparable to a tumor-suppressor gene [11]. SIRT3 expression is increased in breast cancer [12] and is also implicated in the basal apoptotic process, although it is less relevant than SIRT1 [13]. To date, these and other observations allow us to state that SIRTs have a dual role in cancer, acting as either oncogenic or oncosuppressor factors on the basis of the cellular makeup, confirming their fundamental modulatory function in cancer development [1,14].

**Figure 1:**
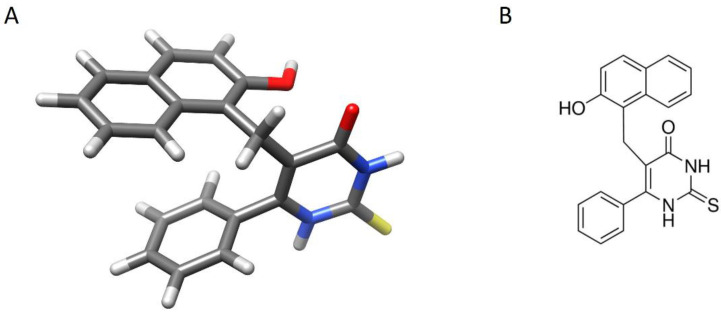
Structure of heterocyclic compound cambinol. (**A**): Three-dimensional view of cambinol. Atoms are colored with conventional code (gray: carbon; white: hydrogen; blue: nitrogen; red: oxygen; yellow: sulfur). The image is generated with UCSF Chimera software (see Methods for references). (**B**): chemical structure of cambinol in 2D.

Transcriptional dysregulations, related to numerous human diseases, are due to alterations in the equilibrium of HAT/HDAC that so constitute the potential target of new drugs such as the HDAC inhibitors (HDAC-I). NAD^+^-dependent HDACs belonging to class III (sirtuins) are not inhibited by conventional HDAC-I such as TSA and SAHA [15]. Among the sirtuin inhibitors, we can list nicotinamide (formed by the deacetylation reaction), sirtinol, M15, and splitomycin [2,16]. However, the rapid hydrolysis of splitomycin and related lactone compounds at neutral pH (half-life 30 min at pH 7.4) makes their therapeutic use difficult in mammalian cells. Being a precursor for NAD^+^, nicotinamide non-selectively inhibits SIRTs through competition to the NAD^+^ binding site [17]. The development of novel agents that specifically block sirtuin activity could provide a therapeutic strategy for the treatment of numerous diseases. Heltweg et al. (2006) [18] identified and characterized cambinol (Figure 1), a chemically stable synthetic heterocyclic compound, which shares the pharmacophore β-naphthol with sirtinol and splitomycin (the chemical structures of these inhibitors are reported in Supplementary Figure S1).

Cambinol showed in vitro inhibitory activity of the human deacetylases SIRT1 and SIRT2, with IC_50_ values of 56 and 59 μM, respectively. Cambinol had weak inhibitory activity against SIRT5 (42% inhibition at 300 μM) and no activity against SIRT3. Class I and II HDACs are not sensitive to inhibition by cambinol, while SIRT4, SIRT6, and SIRT7 do not possess in vitro deacetylase activity. Enzymatic activity studies using NAD^+^ cofactor and acetyl-histone H4-peptide as substrate of sirtuins showed that cambinol is a competitive inhibitor toward H4-peptide, but not toward NAD^+^ [18]. Therefore, cambinol does not directly interfere with the Rossman fold and the NAD^+^ binding to sirtuins. This evidence suggests that it does not affect other NAD^+^-dependent enzymes, such as dehydrogenases, specifically blocking the sirtuin activity. It is this peculiarity that makes cambinol a drug candidate with potentially reduced adverse effects in the therapeutic use.

More recently, new research is revealing the molecular interactions in the sirtuin–inhibitor complexes and the underlying mechanisms of how these inhibitors have an impact on cell growth and differentiation [19]. Therefore, in the present study, we decided to investigate the effects of the sirtuin inhibitor cambinol on the induction of cell differentiation in vitro, and to study the molecular mechanisms of interaction of cambinol with SIRTs in silico. In more detail, we analyzed the cell differentiation activity of cambinol on tumor and normal cell lines. Then, we focused our studies to characterize the differentiating effect on the 3T3-L1 murine embryonic fibroblast cell line, which is potentially able to mature into adipocytes under appropriate conditions [20]. Our results demonstrated that cambinol was able to differentiate 3T3-L1 pre-adipocytes into mature adipocytes, also inducing important cell cycle inhibitors (p16, p27, p130) and the expression of nuclear receptors such as retinoic acid receptors (RARs) and PPARγ. Moreover, we explored the action mechanism of cambinol on human SIRT1 and 2 performing in silico molecular simulations by protein–ligand docking. The 3D molecular models showed that cambinol is able to interact with the substrate-binding site of SIRT1 better than that of SIRT2.

## 2. Materials and Methods

### 2.1. Cell Line Culture, Treatment Conditions, and Differentiation Assay

The 3T3-L1 mouse cell line [20] and MCF-7 breast cancer cell line were obtained from American Type Culture Collection (ATCC, Rockville, MD, USA). Cells were grown at 37 °C with 5% CO_2_ atmosphere in formulated Dulbecco’s Modified Eagle’s Medium (DMEM; Thermo Fisher Scientific, Carlsbad, CA, USA), completed with bovine calf serum to a final concentration of 10%, 1% L-glutamine, 1% ampicillin/streptomycin, and 0.1% gentamicin. Human acute promyelocytic leukemia NB4 cell line, provided by M. Lanotte (INSERM U-496, Centre G. Hayem, Hospital Saint-Louis, Paris, France), was grown at 37 °C in 5% CO_2_ atmosphere in RPMI-1640 medium (Gibco, NY, USA), supplemented with 10% heat-inactivated bovine calf serum, 1% L-glutamine, 1% ampicillin/streptomycin, and 0.1% gentamicin. The cell lines were treated for 5 days (3T3-L1 and MCF-7) and 7 days (NB4) with 50 μM cambinol (100 mM in DMSO, Merck Life Science, Milano, Italy). Cell morphology was analyzed after staining by Oil Red O solution (Merck Life Science) for 3T3-L1 and MCF-7 cells, and after staining by May–Grünwald–Giemsa (Sigma-Aldrich, Saint Louis, MO, USA) for NB4 cells. Then, 3T3-L1 cells were treated with cambinol to a final concentration of 50 μM, with a drug cocktail consisting of troglitazone (5 µM), insulin (1 µg mL^−1^), and dexamethasone (1 µM), and their combination cocktail–cambinol for the time periods indicated in each experiment. After the treatments as above, 3T3-L1 cells were analyzed for adipogenic differentiation by Oil Red O staining following the protocol reported in Bontempo et al. (2015) [21].

### 2.2. Cell Cycle Analysis and Evaluation of Pre-G1 Phase

For cell cycle analysis and evaluation of pre-G1 phase, samples were processed and analyzed as previously reported by Bontempo et al. (2013) [22]. Briefly, 3T3-L1 cells were plated (2 × 10^5^ cells/mL) and then collected after the treatments, centrifuged, and resuspended in PBS 1× containing propidium iodide (PI, 50 μg/mL), sodium citrate (0.1%), and NP40 (0.1%). Cell cycle was analyzed using the FACScalibur flow cytometer with ModFit technology (Becton Dickinson) and cell death levels were measured as pre-G1 DNA fragmentation.

### 2.3. Western Blot Analyses

Cell lysis was carried out as previously reported [23]. After centrifugation of cell lysate at 13,000× *g* for 30 min, protein concentration was determined, and protein extract aliquots of 40 µg were separated by 12% polyacrylamide gel electrophoresis and blotted as previously described [23]. Western blots were run for p16, p27 using mouse monoclonal antibodies (dilution 1:500; #sc-1661; #sc-1641; Santa Cruz Biotechnology-SCBT, Santa Cruz, CA, USA); for Rb130 using rabbit polyclonal antibodies (dilution 1:500; #sc-317; SCBT); immunodetection was performed for RARα, RARβ, and RARγ proteins (retinoic acid receptors) using rabbit polyclonal antibodies (#sc-551; #sc-552; #sc-550; SCBT) with a dilution of 1:500; for the PPARγ protein using a mouse monoclonal antibody (#sc-7273; SCBT) with a 1:500 dilution; for acetylated histone H3 (Lys27) using a rabbit polyclonal antibody (#4353; Cell Signaling Technology—CST, Europe, B.V., Leiden, The Netherlands) with a dilution of 1:500; for acetylated α-tubulin protein using a mouse monoclonal antibody (#MABT868; Merck KGaA, Darmstadt, Germany) with a 1:500 dilution; for acetyl-p53 (Lys373, Lys382) using a polyclonal rabbit antibody (#06-758, Merck) with a 1:500 dilution; for SIRT2 using a polyclonal rabbit antibody (#LS-B3221, LS-Bio, Seattle, WA, USA) with a 1:500 dilution; and ERKs (with rabbit polyclonal antibody, #sc-94; SCBT, dilution 1:1000) was used to normalize the samples for equal loading. Immunoprecipitation (IP) was performed to analyze PPARγ acetylation. 3T3-L1 cells treated and untreated with 50 µM cambinol were lysed in TAP buffer (Tris-HCl pH 7.0, 50 mM NaCl 180 mM, NP-40, 0.15% glycerol, 10% MgCl_2_, 1.5 mM NaMO_4_, 1 mM NaF) with protease inhibitors (Sigma), 1 mM DTT, and 0.2 mM PMSF. Briefly, 650 µg was pre-cleared with 20 μL A/G Plus Agarose (#sc-2003; SCBT). Mouse anti-PPARγ (#95128; CST) or purified mouse IgG (#sc-2025; SCBT) antibodies were added and IP proceeded overnight at 4 °C. Then, 30 μL A/G Plus Agarose was added and incubated for 2 h. Finally, 20 μL of 2× concentrated electrophoresis sample buffer was added. Following Western blot analyses, mouse anti-PPARγ (#95128; CST; 1:500 dilution), mouse anti-acetyl-Lysine (#05-515; Sigma-Aldrich; 1:500 dilution), and normal mouse anti-IgG (#sc-2025; SCBT; 1:500 dilution) were used. Secondary antibodies (CyDye™ 800; #GE29360790; #GE29360788; GE Healthcare-Amersham, Biosciences, Milano, Italy) were used with a dilution of 1:10,000. At the end, the target proteins were visualized by chemiluminescence (ECL Kit; GE Healtcare-Amersham, Biosciences) and semi-quantified by densitometry using the Java-based image-processing and analysis software ImageJ (U.S. National Institutes of Health, Bethesda, MD, USA).

### 2.4. Molecular Docking Simulations and Sequence Comparison

In order to analyze the interactions between cambinol and SIRTs, molecular docking studies were performed. Three-dimensional structures of SIRT1, 2, and 3 available in Protein Data Bank (PDB) [24] were analyzed to select the ones with the best overall quality and suitable conformation features. Particularly, the structures with PDB codes 4I5I [25], 5DY4 [26], and 4BN5 [27] were selected for SIRT1, 2, and 3, respectively. The X-ray structures of SIRT1 and 3 have 2 and 12 chains, respectively, but since they are monomeric proteins, we decided to only perform our study on chain A. All structures presented an inhibitor co-crystallized with the protein, i.e., EX527 analog for SIRT1, SirReal for SIRT2, and SRT1720 for SIRT3. The NAD^+^ cofactor was present for SIRT1 and 2, while Carba-Nicotinamide-Adenine-Dinucleotide (CNA) was present for SIRT3. Since it is known from the literature that the binding to the inhibitor causes an important movement in the NAD^+^ molecule, blocking its kinked active conformation [25], molecular docking simulations focused on the active site were performed, which enabled flexibility of NAD^+^ for SIRT1 and 2 and of CNA for SIRT3. Water molecules present in the active site of SIRT1 and 2 crystals were kept in order to study their possible functional role in the inhibitor binding. Redocking with their respective inhibitors and exploiting blind and focused rigid body docking procedures were also performed, according to the procedure in use in our laboratory and similar studies from the literature [3,28]. For better system control, docking simulations in the presence or absence of the cofactor and of the water molecules were performed, both focused and blind. Both rigid and flexible docking were executed by AutoDock 4.2.5.1 [29], proteins and ligand were prepared by AutoDock Tools 1.5.6 [29], and docking results analyzed with the same. The .pdbqt formats of the complexes were converted to .pdb using OpenBabel 3.1.1 [30]. Parameters applied for SIRT1 docking are as follows: grid box 82 × 126 × 110 and spacing 0.514 Å (grid center: 42.573, −22.315, and 21.547) for blind docking, and grid box 62 × 80 × 56 and spacing 0.375 Å (grid center: 44.926, −22.087, and 22.662) for focused docking. Parameters applied for SIRT2 docking are as follows: grid box 118 × 108 × 126 and spacing 0.514 Å (grid center: −14.461, −26.079, −0.222) for blind docking, and grid box 92 × 80 × 78 and spacing 0.375 Å (grid center: −15.981, −23.675, 10.895) for focused docking. Parameters applied for SIRT3 are as follows: grid box 126 × 100 × 126 and spacing 0.460 Å (grid center: 225.093, 12.431, 20.687) for blind docking, and grid box 76 × 82 × 68 and spacing 0.375 Å (230.228, 4.936, 13.084) for focused docking.

The images of molecular structures were generated using the software UCSF Chimera [31] or AutoDock Tools [29], as reported in each figure legend. Sequence alignment was obtained by Clustal O (1.2.4) [32].

## 3. Results and Discussion

### 3.1. Cambinol Induces Cell Differentiation

**Figure 2:**
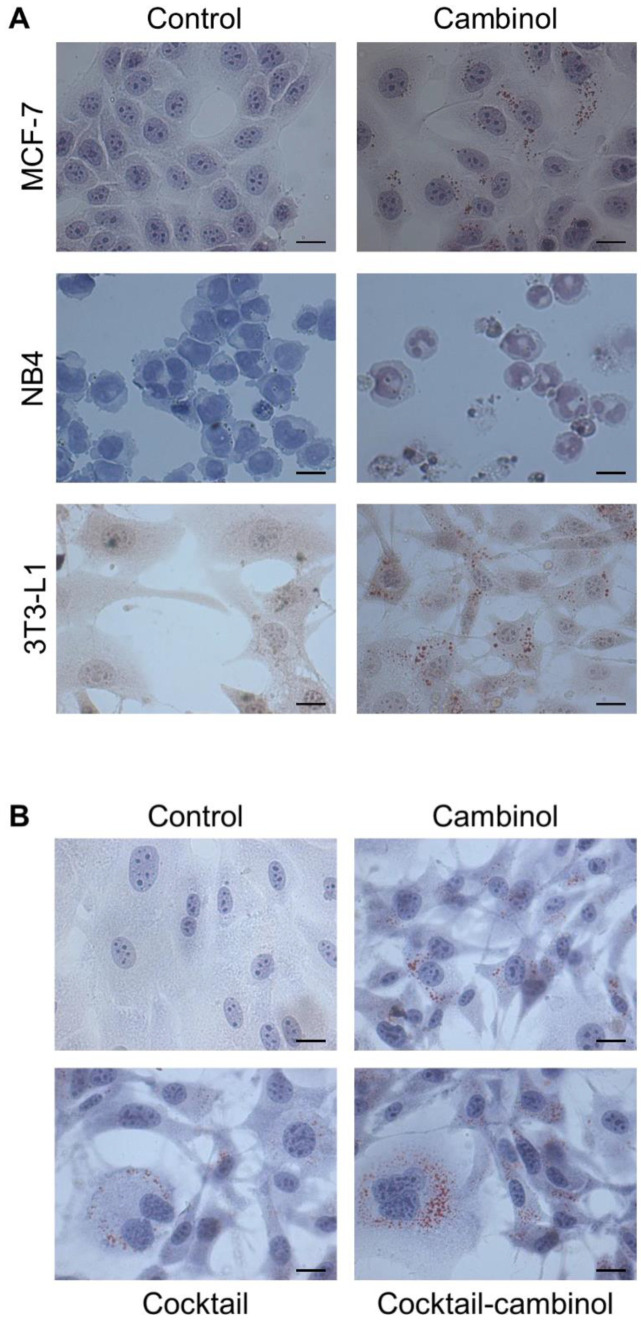
Cambinol induced cell differentiation in the MCF-7, NB4, and 3T3-L1 cell lines. Cells were treated for 5 days (MCF-7 and 3T3-L1) and 7 days (NB4) with cambinol, and then they were stained by Oil Red O and May–Grünwald–Giemsa, respectively (**A**). Cambinol, a drug cocktail, and a cambinol–cocktail combination induced cell differentiation in the 3T3-L1 cell line. Cells were treated for 5 days and then were stained by Oil Red O (**B**). Cells were observed by optical microscopy; size bars: 10 µm.

In this study, the differentiating action of cambinol was initially evaluated with a preliminary morphological screening in the solid and hematological tumor cell lines MCF-7 and NB4, respectively. Cambinol-associated effects, whose antiproliferative and proapoptotic actions are already known in the tumor cell lines, were evaluated in comparison to the 3T3-L1 non-tumor cell model. Interestingly, we found in all three cell lines a differentiating effect after treatment with 50 μM cambinol (Figure 2A). MCF-7, NB4, and 3T3-L1 cells incubated with cambinol showed features resembling the mature cell phenotype. MCF-7 showed features reminiscent of mature mammary phenotypes, including a massive accumulation of neutral lipids, which are an important milk component and the most typical trait of mature epithelial mammary cells. Granulocytic differentiation was observed in NB4 cells of human promyelocytic leukemia. 3T3-L1 cells, a model of potential adipocyte maturation, also responded to the treatment with evident droplets of intracellular lipids. Then, we chose to use the non-tumor cell model 3T3-L1 to investigate how molecular targets are involved in the observed effects under physiological-like conditions. For this purpose, we used a drug cocktail as the positive control, whose differentiating action on the same cell line was already known [20,33]. We evaluated whether it acted with different timing and biological efficacy compared to cambinol. In addition, the cambinol–cocktail combined treatment was applied for finding the existence of potential synergistic or antagonistic effects. Since these inhibitors perform multiple functions, we focused our studies on characterizing their differentiating effects.

Successively, 3T3-L1 cells were separately treated with 50 μM cambinol, cocktail of drugs (5 μM troglitazone, 1 μg mL^−1^ insulin, and 1 μM dexamethasone) whose differentiating effects have already been reported [20,33], and a cambinol–cocktail combination. The results of the optical microscopy observations showed the cell differentiation effect of cambinol, as evaluated by Oil Red O staining (Figure 2B and Supplementary Figure S2). Interestingly, cambinol-induced differentiation emerged more rapidly than that of the cocktail of drugs; in fact, clearly evident lipid droplets appeared in 3T3-L1 cells treated with cambinol already after 2–3 days (data not shown), and only after about 5 days if treated with the cocktail alone (Figure 2B). In the same Figure 2B, it can be seen that a more evident effect was present after 5 days of cambinol administration in combination with the drug cocktail (see also Supplementary Figure S2). The number of Oil Red O positive cells for the presence of lipid droplets is almost comparable after 5 days of treatment with the cambinol or cocktail. However, an important difference can be observed due to the larger size of the lipid droplets in the treatment with cambinol, confirming what has already been said, because the differentiating effect was induced earlier.

### 3.2. Cambinol Modulates Cell Cycle in 3T3-L1 Line

**Figure 3:**
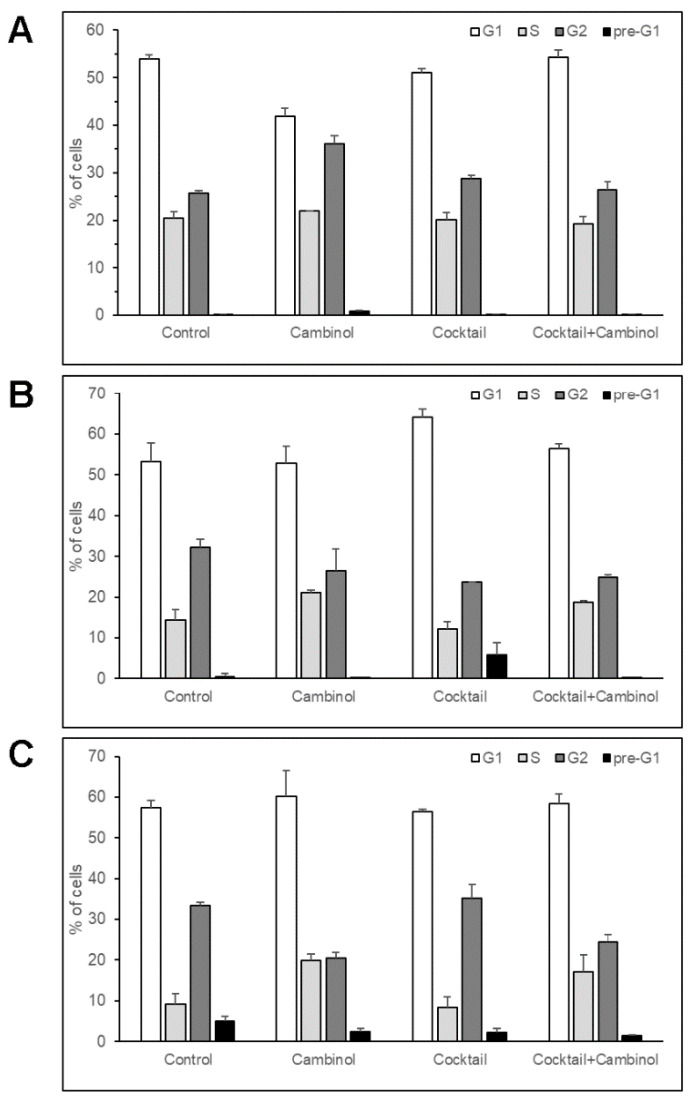
Cell cycle analysis of 3T3-L1 cells treated with cambinol, drug cocktail, and cocktail–cambinol combination for 1 (**A**), 2 (**B**), and 3 days (**C**). The results are reported as the mean ± SD of three independent experiments.

Therefore, with the aim of characterizing the cell differentiation action induced by cambinol, we assayed its biological effects on the cell cycle. For this purpose, the 3T3-L1 cells were treated for 1, 2, and 3 days with 50 µM cambinol, a drug cocktail (5 µM troglitazone, 1 µg mL^−1^ insulin, and 1 µM dexamethasone), and a cocktail–cambinol combination. The analysis of the cell cycle showed that cambinol did not exhibit cytotoxic effects, as detected by the low percentage value of cell death in the pre-G1 phase (for details, see Materials and Methods; Figure 3).

In addition, interestingly, FACS analysis showed that cambinol determined an early and significant increase (41%) of the cell number in the G2-phase of the cell cycle after 1 day of treatment, associated with a reduction (22%) in the cell number in the G1-phase (Figure 3A). This trend was progressively lost in the following 2 days (Figure 3B,C), when a reduction (39%) in the cell number in G2-phase was observed. Overall, the number of cambinol-treated cells decreased in the G2-phase after 3 days as compared to the previous time points, and it also diminished with respect to the other two treatments and to the untreated control (Figure 3C). At the same time, the number of S-phase cells underwent a progressive increase compared to the control up to 3 days in the samples treated with cambinol alone (116%) and with cambinol in combination with the drug cocktail (86%), even if their relative values with respect to the other cellular phases remained approximately constant. The S-phase cells did not undergo significant changes over time (2 and 3 days) with the drug cocktail alone, remaining approximately at the same levels as the control. The observable levels of cell death were negligible with respect to the control in almost all cases, except for the cocktail at 2 days, in which an increase in the cells in the pre-G1 and in the G1 phases was observed, which is associated with a reduction in the number of cells in the G2 phase. This trend is in line with the restoration of the cell differentiation program [21], as also evidenced by the early accumulation of cytoplasmic lipid droplets after 2–3 days, as reported above.

### 3.3. Molecular Targets Involved in the Differentiation Action of Cambinol

In order to tentatively characterize from a molecular point of view the differentiation activity induced by cambinol under physiological-like conditions of 3T3-L1 cells, a model of adipocyte maturation, we analyzed the expression of various protein factors that can play a key role in the control of the cell cycle and in the induction of differentiation. We evaluated the expression of the regulatory cell cycle proteins p16 and p27, both known to cause cell cycle arrest through the inhibition of cyclin-dependent kinases (CDKs) [22,34].

Overall, as shown in Supplementary Figure S3A, these first results seem to demonstrate that cambinol can contribute to modulating the cell cycle progression early after 2–3 days of treatment by up-regulating the expression of p16 and p27, whose functions as cell cycle regulators and tumor suppressors are well-known [34]. Then, cambinol may interact with the drug cocktail by increasing the final effects on both proteins p16 and p27.

Several nuclear receptors are involved in the cell differentiation process, including the retinoic acid receptors (RARs), which are ligand-controlled transcription factors functioning as heterodimers with retinoid X receptors (RXRs) to finely regulate cell growth, differentiation, survival, and death in different cell systems after activation by retinoic acid (Ra) binding [35]. Once inside the cell, Ra is transported through specific cellular transport proteins and, after binding to the specific receptors, can regulate the gene expression through the recruitment of transcription co-activators (such as p160). The immunoblot of protein extracts obtained from 3T3-L1 cells treated with cambinol highlighted an increase in the expression levels of three RARs. In particular, a prolonged effect of cambinol was observed on the expression of RARα, RARβ, and RARγ, at the considered time points, as shown in Supplementary Figure S3A. These first results showed that the treatment with cambinol could also induce the modulation of the expression of these important nuclear receptors related to cell differentiation.

Another important member of the nuclear receptor superfamily of ligand-dependent transcription factors is PPARγ, whose role in adipose tissue differentiation has been extensively studied [36]. The PPARs and the RXRs coordinately regulate gene expression. PPARγ directly regulates gene transcription through the formation of functional heterodimers with RXR and binding to specific DNA sequences (PPAR response elements) [37]. In this way, PPARγ can regulate the storage of fatty acids and glucose metabolism by intervening in the adipogenesis process. PPARγ has been identified to possess different isoforms, differentially transcribed from the same gene. Among these, PPARγ1 is ubiquitous, but highly expressed in adipose tissue, while PPARγ2 (with an additional 30 amino acids on N-terminal of PPARγ1) is exclusively expressed in adipose tissue. A high adipogenesis capacity is the main function of PPARγ2. Since it was reported that cambinol can modulate the activity of PPARγ [38], to further characterize its cell differentiation activity, we thought of studying the possible involvement of PPARγ by analyzing the expression levels of this receptor by immunoblotting. Our results, obtained by treating 3T3-L1 cells with cambinol, indicated that adipocyte differentiation was induced even in the absence of troglitazone (Figure 2), a ligand agonist of PPARγ [39]. As shown in Supplementary Figure S3A, the first results seem to indicate that cambinol induces adipogenesis through the PPARγ activation route, working synergistically with the drug cocktail. In conclusion, the expression of full-length PPARγ and then its cleavage are essential for cell differentiation [36,40,41].

The retinoblastoma tumor-suppressor family proteins (pRb) are key factors in the control of cell proliferation and are also known as “pocket proteins” [42]; their overexpression induces cell proliferation arrest. We analyzed the expression levels of Rb130 by immunoblotting and found an increased expression level after 3 days and up to 5 days after treatment with cambinol and with cambinol together with the drug cocktail (Supplementary Figure S3B). These first results seem to indicate that the cell differentiation effect of cambinol also involved the Rb130 protein, which is related to cell differentiation, as well as being notoriously up-regulated in differentiated cells [42]. The evidence indicated that pRb bind a variety of transcription factors and chromatin remodeling enzymes, forming transcriptional repressor complexes that control gene expression [43]. More recently, Li et al. (2018) [44] demonstrated that the activation of the Rb pathway in a transient manner is important for cell differentiation. Therefore, cambinol could play an important role in regulating differentiation capacities through Rb up-regulation.

It is well-known that cambinol acts as an inhibitor of NAD^+^-dependent deacetylases (class III HDAC or sirtuins) by specifically modulating the activity of the human SIRT1 and SIRT2 [18]. Therefore, we wanted to verify if in our conditions the differentiation effect could be correlated to a possible epigenetic modulation exerted by the cambinol itself. To this aim, we evaluated the variation of the acetylation status of histone and non-histone targets. In our study, histone H3 was analyzed, and the results showed an increase in its acetylation levels upon cambinol treatment (data not shown). To evaluate the acetylation status of non-histone targets, the proteins α-tubulin and p53 were chosen. The results of immunoblotting showed increased protein acetylation at 2 days for both p53 and α-tubulin in cambinol-treated samples as compared to the untreated control (data not shown). SIRT1-dependent PPARγ deacetylation is known to be an important and selective modulation of PPARγ action [45]. PPARγ is considered a lipid sensor that, when activated by acetylation, can stimulate gene expression, promoting lipid accumulation and storage [37,46,47]. Our further experiments also detected an increase in the PPARγ acetylation levels after 2 days of cambinol treatment (data not shown) modulating its own activity. In the following analyses, we also studied the variation of expression levels of the SIRT2 protein. The results, shown in the Supplementary Figure S4, indicated unchanged levels of SIRT2 protein expression after 2 days of cambinol treatment. This confirms that the observed acetylating effect of cambinol was due to the well-known inhibition of the deacetylase activity of SIRTs, not associated with the variation of their expression. All this allows us to state that the differentiating action may in part be clearly related to the cambinol-induced hyperacetylation of non-histone and histone cell targets due to sirtuin inhibition. Furthermore, in-depth studies are needed in this regard on the same and other representative targets of adipogenesis to confirm and better investigate these aspects at the molecular level.

### 3.4. Docking Simulations of Molecular Interactions between Cambinol and the Target SIRTs

Since the observed effects of cambinol are the result of sirtuin inhibition, it was interesting to explore the inhibitory mechanism of cambinol on human SIRT1, 2 and 3 in silico by protein–ligand docking simulations.

**Figure 4:**
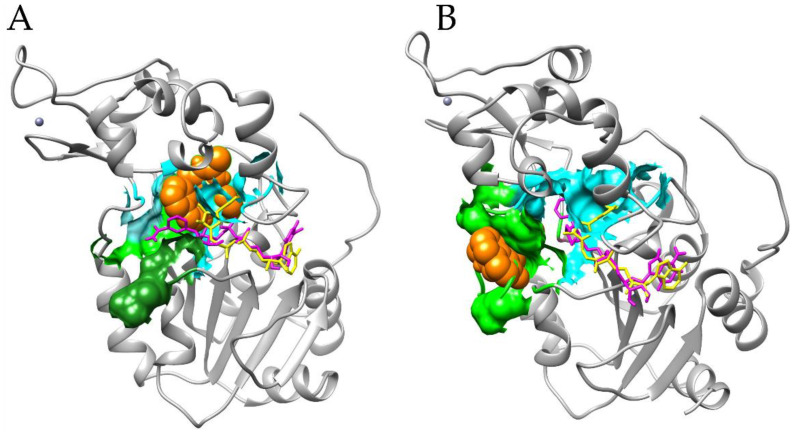
Cambinol binding to the canonical inhibition pocket (**panel** (**A**)) and to the substrate site (**panel** (**B**)). SIRT1 is represented in gray cartoons, the Histone H4 peptide in dark green spheres, the Carba-Nicotinamide-Adenine-Dinucleotide (CNA) and Nicotinamide-Adenine-Dinucleotide (NAD^+^) in yellow and fuchsia ball and sticks, respectively; cambinol is in orange spheres; and the indole (EX527 analogue) SIRT1 inhibitor pocket and the Histon binding site in cyan and light green surfaces, respectively. Cambinol binding to both binding pockets forces the NAD^+^ in a stretched conformation that does not allow the correct interaction of the histone in the pocket, for which an NAD^+^ kinked conformation is necessary (represented as an example by the CNA) [25]. In (**panel** (**A**)), in fact, cambinol covers part of the kinked NAD^+^ binding site (note steric clashes between CNA and cambinol); in (**panel** (**B**)), instead, it makes direct interaction with stretched NAD^+^ conformation. Moreover, this conformational change determines steric clashes between NAD^+^ and the substrate, preventing the correct interaction of the latter with the active site and the cofactor; moreover, the presence of cambinol in the binding site represents a hindrance to the peptide binding. The image is generated with UCSF Chimera software (see Methods for references).

Docking experiments performed on SIRT1 show that cambinol seems to prefer a non-canonical inhibition site, moving into or behind the site for access to the substrate. In particular, docking with flexible NAD^+^ shows a difference of about 1 Kcal/mol between the binding to the substrate site (lowest binding energy equal to −7.29 Kcal/mol) and to the canonical inhibition pocket (lowest binding energy equal to −6.35 Kcal/mol). Better values are obtained for the non-canonical site under other docking conditions (see Table 1 and Figure 4).

However, both the binding poses unveil the blocking of the NAD^+^ kinked active conformation, which results in a stretched conformation. Docking performed with a rigid NAD^+^ in inhibited conformation and functional water molecules shows a similar binding energy (−7.57 Kcal/mol). It is important to underline that the redocking analysis, performed exploiting the inhibitor co-crystallized with the protein structure selected, showed a better binding energy (−11.25 Kcal/mol) for binding to the canonical inhibition cavity, but a worst binding energy for the site of the substrate access (−6.44 Kcal/mol) (Table 2). These results suggest that cambinol is able to act on SIRT1 in a different mechanism compared to the co-crystallized inhibitor.

**Figure 5:**
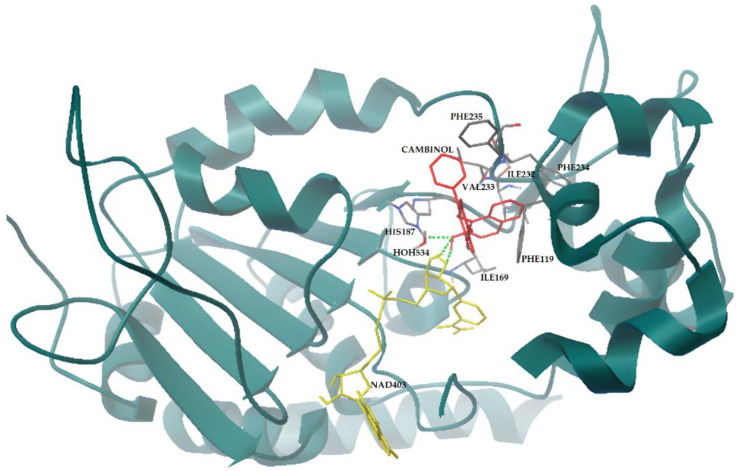
Cambinol and SIRT2 interactions resulting from flexible docking simulation. Cambinol (in red sticks) forms H-bonds with the cofactor and the active site H187 (green dots), inhibiting the enzyme. SIRT2 is represented by dark green cartoon; NAD^+^ and SIRT2 residues involved in the interactions are represented by yellow and gray sticks, respectively. The image is generated using AutoDockTools software (see Methods for references).

In the case of SIRT2 and cambinol, the results of both docking approaches, i.e., whether flexible or rigid NAD^+^ with functional water molecules display a higher propensity of cambinol to occupy the same position detected for the co-crystallized inhibitor, i.e., the inhibition site. This finding is in agreement with previous work that simulated an interaction between cambinol and SIRT2 [47]. The binding energies are comparable, too, with the lowest binding energy that varies from −9.71 to −9.33 Kcal/mol for the cambinol under the different docking conditions (see Table 1) and from −12.39 and −10.92 Kcal/mol for the SirReals inhibitor (see Table 2). In a docking simulation with flexible NAD^+^, the binding energy value at the inhibition site is very similar (−9.35 Kcal/mol). However, the position is slightly shifted toward the substrate binding site, with a partial occlusion of its access. This finding confirms the experimental results reported in the literature at the molecular interaction level [18], which indicates that cambinol is competitive with substrates. Cambinol makes a direct interaction with NAD^+^, forming H-bonds with the cofactor and inhibiting its correct conformation (Figure 5). Moreover, it also forms an H-bond with the active site H187, preventing its activity; a water molecule seems to also be involved in the binding of the inhibitor. The partial occlusion of the substrate binding site seems to also be detected by re-docking for the already known inhibitor (data not shown). Moreover, docking performed without NAD^+^ did not reveal a cambinol competition with the cofactor (data not shown), which is in agreement with the literature [18].

**Figure 6:**
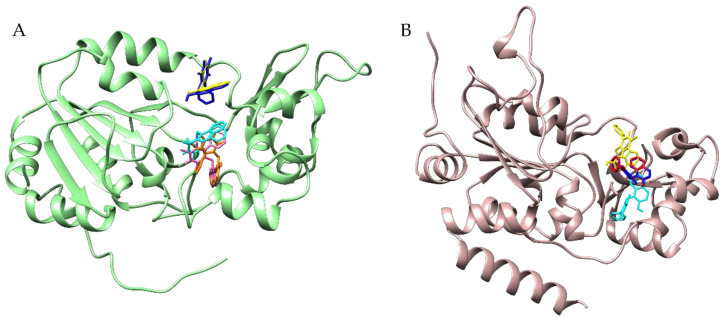
Cambinol binding to the SIRT1 (**panel** (**A**)) and to SIRT2 (**panel** (**B**)) using different docking protocols. SIRT1 is in green cartoons, while SIRT2 is in rosy cartoons; the cambinol is in different color sticks. More specifically, in (**panel** (**A**)), cambinol is represented in blue and magenta for the poses detected by focused docking, including NAD^+^ but not water molecules; in cyan for the poses detected by focused docking without NAD^+^ and water; and in yellow and orange for the poses detected by focused docking, including NAD^+^ and water molecules. In (**panel** (**B**)), instead, cambinol is highlighted in red for the best pose from focused docking including NAD^+^ but not water molecules, in cyan for the pose from focused docking without NAD^+^ and water, and in yellow and blue for the poses from the focused docking including water molecules and flexible and rigid NAD^+^, respectively.

Particularly, docking performed for SIRT1 and SIRT2 in the absence of water molecules and a cofactor (Supplementary Table S1) confirmed the detection of the same binding pockets obtained in the presence of these compounds. All the tested conditions (i.e., docking in the presence of NAD^+^ and water molecules in the active site, docking in the presence of the cofactor but without water molecules, and docking without all heteroatoms) confirmed for SIRT1 the possibility of cambinol to bind to both the inhibition and substrate binding sites, and for SIRT2 the preference for the inhibition site (Figure 6).

The docking simulations of the interaction of cambinol with SIRT3 show that, under blind conditions, neither the inhibition site nor the binding site are identified as energetically preferred, and the ligand is positioned on the protein surface without specific preferences. In particular, the binding site of the co-crystallized inhibitor was not recognized at all as a possible binding site for cambinol. Focused docking between SIRT3 and cambinol with NAD^+^ shows that cambinol might recognize the substrate binding site. When NAD^+^ is kept rigid, the predicted energy is −7.36 Kcal/mol, while in the simulations performed with a flexible NAD^+^, the predicted energy is −8.12 Kcal/mol (see Table 1). When NAD^+^ is not present in the crystal, cambinol does not recognize the enzyme binding site and positions itself on the surface of the protein (data not shown).

By comparing the results of the molecular docking of cambinol with the three SIRTs (see Table 1), the best binding energy values are obtained for SIRT2, while the values with SIRT1 and SIRT3 are similar. However, the redocking simulations with the co-crystallized inhibitors must also be considered. Although the predicted binding energies indicate that cambinol could bind to SIRT3, the values may be not enough to inhibit enzymatic activity, as redocking of SIRT3 with its inhibitor predicted values of about −13 Kcal/mol (see Table 2). The energy difference of about 6 kcal/mol between redocking and SIRT3-cambinol docking is higher than SIRT1 (about 4 kcal/mol) and SIRT2 (about 2–3 kcal/mol) simulations, thus explaining in part why cambinol does not inhibit SIRT3 [18].

**Figure 7:**
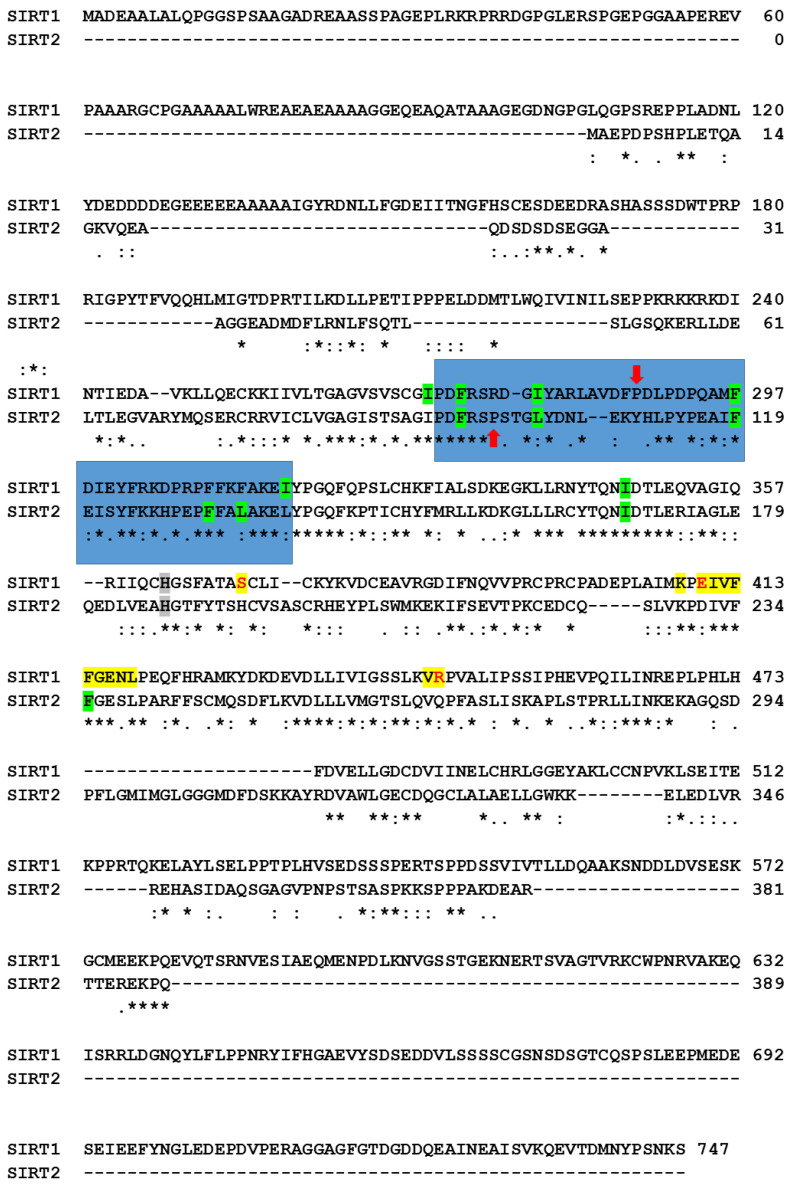
Alignment of SIRT1 and SIRT2 sequences. Amino acids of SIRT1 at the substrate binding site that interact with cambinol at different conditions are highlighted in yellow. The red letters evidence the three amino acids (i.e., Ser370, Glu410, and Arg446) without identical correspondence in SIRT2. Amino acids of SIRT1 and SIRT2 at the inhibition site that interact with cambinol at different conditions are highlighted in green. Histidines of the catalytic site are represented by gray background. The 271-316 region of SIRT1 and 94-138 region of SIRT2, which are part of the inhibition sites, are evidenced by the blue background. Red arrows indicate the two prolines without correspondences in the other sequence (i.e., Pro288 in SIRT1; Pro99 in SIRT2). Alignment has been obtained by Clustal O (1.2.4; see Methods for references), which adds symbols under the aligned amino acids to evidence identical residues (asterisk), strong similarity (colon), or similarity (period).

Since the docking results indicate a preference of cambinol for the substrate binding site over the inhibition site only in the case of SIRT1, we investigated the detail of the residues at the interface, as reported in the last column of Table 1. Two aromatic residues of SIRT1 are involved in both sites (Phe413 and Phe414 in the binding site; Phe273 and Phe297 in the inhibition site). Therefore, in both cases, cambinol finds a similar aromatic environment. However, the inhibition site presents only hydrophobic residues, except for the catalytic His. On the contrary, the substrate binding site also presents polar residues, which may interact with the polar moiety of cambinol and form H-bonds (see the underlined residues in the last column of Table 1). This may explain the preference of cambinol for the substrate binding site instead of the inhibition site in SIRT1. By comparing all SIRT1 amino acids at the substrate binding site in different conditions (Figure 7, residues in yellow) to the aligned corresponding amino acids in SIRT2, we found that most of the amino acids were identical, with the exception of three polar residues of SIRT1, i.e., Ser370, Glu410, and Arg446, substituted in SIRT2 by His, Asp, and Gln, respectively. Although in SIRT2 the amino acids were still polar, the differences in charge state (Ser/His and Arg/Gln substitutions), and length of the side chain (Glu/Asp) may prevent the formation of appropriate interactions, and explain why cambinol prefers to bind at different sites of SIRT1 and SIRT2.

**Figure 8:**
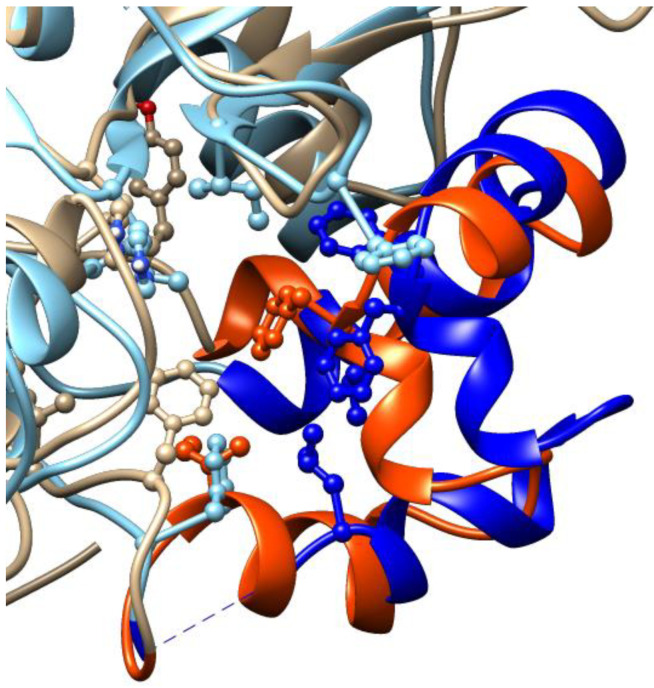
Comparison between the mismatching portions at the canonical inhibitory site of SIRT1 and 2, represented in beige and cyan cartoons, respectively; the SIRT1 region between residues 271–316 is highlighted in orange; the region between residues 94–138 of SIRT2 is shown in blue. Amino acids interacting with cambinol are represented in balls and sticks. The image is generated with UCSF Chimera software (see Methods for references).

Moreover, the comparison of the two sequences indicates a good level of conservation of the residues interacting with cambinol at the canonical inhibition sites (Figure 7, residues in green). However, we note that the regions 271–316 of SIRT1 and 94–138 of SIRT2, which are part of the inhibition site, present relevant differences and sequence gaps. The low similarity of the two regions coincides with the mismatch at the conformational level (Figure 8), which is probably due to the presences of prolines without correspondence in the other sequence (i.e., P288 in SIRT1 and P99 in SIRT2). It is well-known that prolines, due to their imino acid structure, impose irregular conformations of the backbone. It is likely that the conformational difference affects the final inhibition site shape, more favored for cambinol in SIRT2 than SIRT1, as indicated by the binding energy values in Table 1 (around −6 Kcal/mol for SIRT1 and −9 Kcal/mol for SIRT2).

## 4. Conclusions

Altogether, the findings presented here highlight the ability of cambinol to induce the differentiation of 3T3-L1 preadipocytes into mature adipocytes, likely by modulating important nuclear receptors. Furthermore, the results on the identification and characterization of the effects of cambinol suggest that the inhibition of SIRTs can be used as a treatment for human pathologies in which they perform essential functions. The identification of other SIRT targets involved in the pathogenesis of metabolic and tumor pathologies paves the way for the possibility of revealing other contexts where the inhibition of deacetylases represents a real opportunity for a therapy. The ability of cambinol to specifically inhibit some SIRTs makes this synthetic molecule a potential model structure in the research and development of new therapeutic agents that are able to decisively counteract various human pathologies such as tumors and metabolic alterations. Our molecular docking studies, showing that cambinol is potentially able to interfere with substrate binding, indicate possible differences in the mechanism of the inhibition of SIRT1 and SIRT2. However, further studies are necessary to fully understand the molecular mechanisms underpinning the cellular responses and SIRT inhibition for the potential applications of cambinol and its derivatives as candidate drugs.

## Figures and Tables

**Table 1:** Binding energies from docking simulations of cambinol interaction with SIRT1, 2, and 3.

Receptor ^a^	Docking Procedure		Binding Energy (Kcal/mol)	Interaction Region on the Receptor ^b^
SIRT1	Blind		−7.66 ^c^	Near the binding site (Ser370, Lys408, Glu410, Ile411, Val412, Glu416, Asn417, and Leu418)
			−7.57 ^c^	Binding site (NAD, His363, Val412, Phe413, Phe414, Gly415, Glu416, Asn417, Leu418, and Arg446)
	Focused	Rigid NAD^+^	−7.96 ^d^	Near the binding site (Ser370, Lys408, Glu410, Ile411, Val412, Glu416, Asn417, and Leu418)
			−7.91 ^d^	Binding site (NAD, His363, Phe413, Phe414, Gly415, Glu416, Lys444, Val445, and Arg446)
		Flexible NAD^+^	−7.29 ^e^	Binding site (NAD, His363, Val412, Phe413, Phe414, Gly415, Glu416, Leu418, Val445, and Arg446)
			−6.35 ^e^	Inhibition site (NAD, Ile270, Phe273, Ile279, Phe297, Ile316, Ile347, His363, HOH717, and HOH702)
SIRT2	Blind		−9.52	Inhibition site (NAD, Phe96, Leu103, Phe119, Phe131, Leu134, Ile169, His187, Ile232, and Phe234)
	Focused	Rigid NAD^+^	−9.71	Inhibition site (NAD, Phe96, Leu103, Phe119, Phe131, Leu134, Ile169, His187, Ile232, and Phe234)
		Flexible NAD^+^	−9.35	Inhibition site partially occluding the binding site (NAD, Phe119, Ile169, His187, Ile232, Val233, Phe234, Phe235, and HOH534)
SIRT3	Blind		−7.06	Protein surface (Phe157, Arg158, Leu168, Gln169, Gln171, Asp172, Leu173, Tyr175, and Pro176)
	Focused	Rigid CNA	−7.36	Binding site (Phe157, Glu177, Phe180, Val292, Phe293, and Phe294)
		Flexible CNA	−8.12	Binding site (NAD, Glu177, Gln228, Ile230, His248, Phe180, Ile291, Val292, Phe294, and Val324)

^a^ The structures with PDB codes 4I5I [25], 5DY4 [26], and 4BN5 [27] have been selected for SIRT1, 2, and 3, respectively. ^b^ Residues interacting with cambinol are reported in parenthesis; residues forming H-bonds are underlined. ^c^ Two clusters of conformations under blind docking simulation are reported because they have similar energies and different interaction region on the receptor. ^d^ Two clusters of conformations under focused docking simulation with rigid NAD^+^ are reported because they have similar energies and different interaction region on the receptor. ^e^ Two clusters of conformations under focused docking simulation with flexible NAD^+^ are reported because they have different interaction region on the receptor.

**Table 2:** Binding energies from redocking simulations of interaction of SIRT1, 2, and 3with the co-crystallized inhibitors.

Receptor/Inhibitor	Docking Procedure	Binding Energy (Kcal/mol)	Interaction Region on the Receptor
SIRT1/EX527 analog ^a^	Blind	−11.07 ^b^	Inhibition site
		−6.44 ^b^	Binding site
	Focused	−11.25	Inhibition site
SIRT2/SirReal ^a^	Blind	−10.92	Inhibition site
	Focused	−12.39	Inhibition site
SIRT3/SRT1720 ^a^	Blind	−13.66	Inhibition site
	Focused	−13.59	Inhibition site

^a^ The structures with PDB codes 4I5I [25], 5DY4 [26], and 4BN5 [27] have been selected for SIRT1, 2, and 3, respectively. All structures present an inhibitor co-crystallized with the protein. ^b^ Two clusters of conformations under blind docking simulation are reported because they have different interaction regions on the receptor.

## Data Availability

Not applicable.

## Supplementary Materials

The following supporting information can be downloaded at https://www.mdpi.com/article/10.3390/biomedicines11061624/s1. Figure S1: Chemical structures of HDAC inhibitors. Figure S2: Quantitative evaluation of the cell differentiation effect. Figure S3: Cambinol modulated expression levels of p16, p27, retinoid receptors (RARs), PPARγ, and Rb130. Figure S4: Cambinol does not modulate the expression level of SIRT2 protein. Table S1: Docking results in the absence of cofactors and water molecules.
