# Supplementary material for: Sirtuin Inhibitor Cambinol Induces Cell Differentiation and Differently Interferes with SIRT1 and 2 at the Substrate Binding Site
Source: Biomedicines. 2023 Jun 2;11(6):1624. doi: 10.3390/biomedicines11061624 (PMC10295654; doi:10.3390/biomedicines11061624)
Supplement: Supplementary file 1 [file biomedicines-11-01624-s001.zip › biomedicines-2313263-supplementary.pdf]

Supplementary Material

Conventional HDAC-I

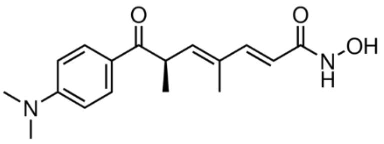

TSA

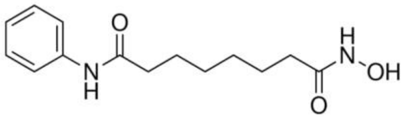

SAHA (vorinostat)

Sirtuin inhibitors

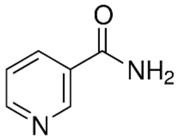

Nicotinamide

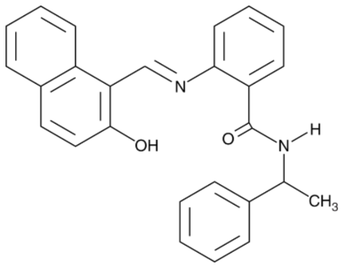

Sirtinol

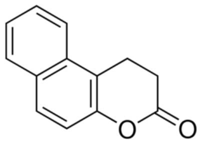

Splitomycin

Figure S1. Chemical structures of HDAC inhibitors (HDAC-I).

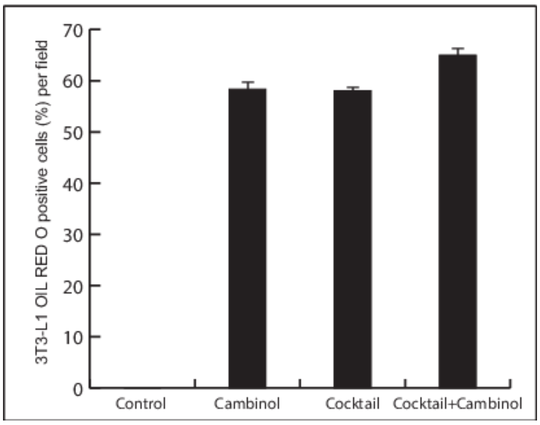

Figure S2. Quantitative evaluation of the differentiation effect on 3T3-L1 cells after 5 days of treatment with cambinol, drug cocktail, and cambinol-cocktail combination compared to the untreated control was expressed as a percentage obtained from the average count of Oil Red O positive cells observed in three different fields under the optical microscope (see also **Figure 2B**). The results are reported as the mean ± SD of three independent experiments.

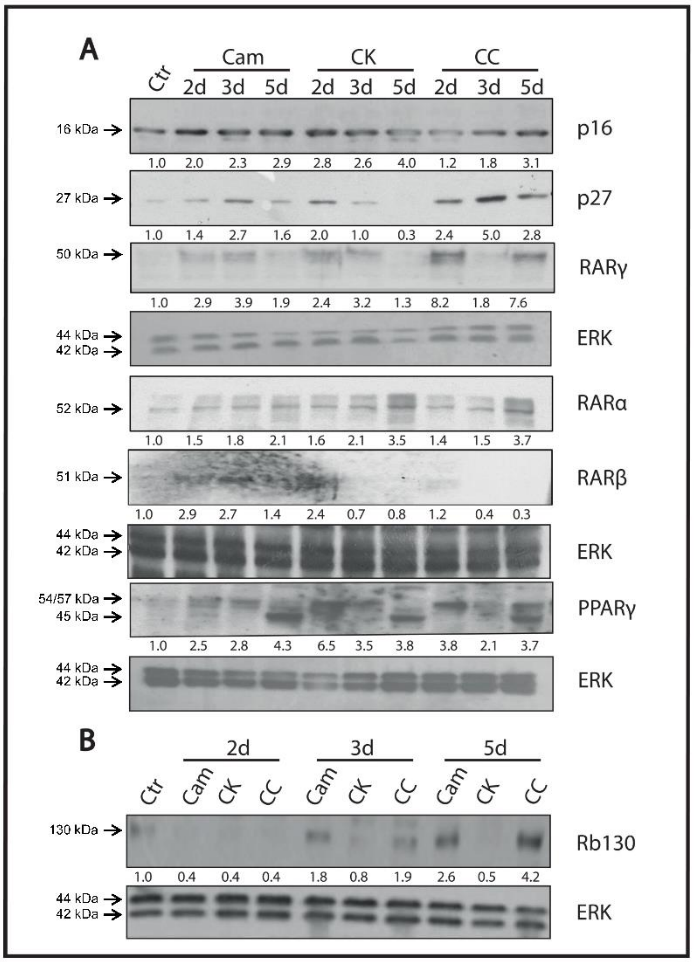

Figure S3. Cambinol modulated expression levels of p16, p27, retinoid receptors (RARα, RARβ, and RARγ), PPARγ (**panel A**) and Rb130 (**panel B**). Immunoblotting of the indicated proteins in cells 3T3-L1 after 2, 3, and 5 days of treatment with cambinol (Cam), drug cocktail (CK), and cambinol+cocktail (CC) with respect to the control not treated (Ctr). ERKs were used as an internal loading control for normalisation of the expression levels. The semi-quantification of protein bands for each lane was performed by densitometry using ImageJ software. Indicative molecular weights were reported where proteins appeared in our Western blot experiments. Arrows pointed to the band of interest under investigation and object of discussion.

Notes to panel A of Figure S3:

p16-p27: The results shown in **Figure S3A** evidenced an increased expression of p16 already after 2 days of treatment with cambinol. The expression levels of p16 were clearly marked over time (up to 5 days), when compared with the effects of the cocktail alone and of the cambinol-cocktail combination. Immunoblots also showed an increased expression level of p27 after 3 days of treatment with cambinol (**Figure S3A**). This effect was also evident after 2 days of treatment with the cocktail alone and the cambinol-cocktail combination, while this latter gave a more sustained response over time (up to 5 days) than the other two treatments. The analysis carried out for the molecular targets p16 and p27 showed that there is a general decrease in the expression levels of these proteins after 5 days of treatment with the cocktail alone. This effect likely occurs because after 2 days of treatment the cell differentiation program is already at an advanced stage, so the presence of these proteins could no longer be necessary. Here, it was interesting to note a long-lasting response of p16 over time with cambinol compared to the treatment with drug cocktail alone, although the combination of cambinol and drug cocktail initially restored the level of control at 2 days. It was possible to evidence a more sustained response of p27 over time with cambinol than with the treatment of drug cocktail alone. Furthermore, interestingly, cambinol showed to modulate the effects of drug cocktail by inverting the expression trends of p16 and p27 in the combined treatment (see CC in **Figure S3A**).

RARs: The results shown in **Figure S3A** evidenced that the expression of RARα with the drug cocktail and cambinol-cocktail showed a trend like to cambinol alone, except for 5 days when there is a more marked level. The effect of treatment with drug cocktail and cambinol-cocktail on RARβ showed an expression increase only at 2 days. The effect of drug cocktail for RARγ at 3 days showed a negative interference with cambinol, while at 5 days there is a synergistic effect of cambinol and cocktail.

PPARγ: An increase in the level of PPARγ1-2 expression already after 2 days of treatment, sustained for 3 days, was observed with cambinol, while at 5 days of incubation we observed the generation of a distinct band at lower molecular weight, likely due to a cleavage of full-length PPARγ, as shown in **Figure S3A**. The presence of this PPARγ-derived fragment was reported to be associated with accumulation of lipid droplets and cell differentiation in 3T3-L1 [40]. More precisely, the truncated PPARγ was clearly detected at 5 days by the appearance of a new band at lower molecular weight concomitant to the decrease of the full-length PPARγ with both the cambinol and cocktail treatments. On the contrary, the expression of full-length PPARγ was markedly increased at 5 days with the cambinol-cocktail treatment associated with the presence of the truncated form of PPARγ.

Note to panel B of Figure S3:

Rb130: The analysis of the Rb130 expression by immunoblotting in **Figure S3B** showed an increased expression level after 3 days and up to 5 days after treatment with cambinol and with cambinol together the drug cocktail.

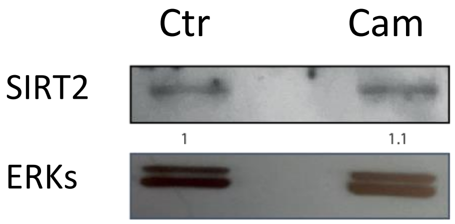

Figure S4. Cambinol does not modulate the expression level of SIRT2 protein. Immunoblotting of SIRT2 protein in cells 3T3-L1 after 2 days of treatment with cambinol (Cam) compared to the untreated control (Ctr). ERKs were used as an internal loading control for normalisation of the protein expression levels.

Supplementary Table S1. Docking results in the absence of cofactors and water molecules. Binding energies from control docking simulations of cambinol interaction with SIRT1 and 2 in presence or absence of cofactor and water molecules.

Receptor	Docking procedure		Binding Energy (Kcal/mol)	Interaction region on the receptor [d]
SIRT1	Blind	NO NAD^+/ NO WATER	-10.20 [a]	Inhibition site (partially)/Binding site (partially) (Ala262, Phe273, Arg274, Tyr280, Gln345, His363, Val412, Phe414, Ser441, Ser442, Val445)
			-9.55 [a]	Inhibition site/Binding site (partially) (Phe273, Arg274, Tyr280, Phe297, Gln345, Asn346, Ile347, His363, Val412, Phe413, Phe414)
		WITH NAD^+/ NO WATER	-9.31[b]	Inhibition site (Ile270, Pro271, Ile297, Ile316, Tyr317, Pro318, Phe273, Ile347, Asp348)
			-7,71[b]	Near the binding site (Ser370, Lys408, Glu410, Ile411, Val412, Glu416, Asn417, leu418, Pro419)
			-7.38[b]	Binding site (NAD, His363, Val412, Phe413, Phe414, Gly415, Glu416, Asn417, Leu418, Arg446)
	Focused	NO NAD^+/ NO WATER	-10.05	Inhibition site (Phe273, Arg274, Tyr280, Phe297, Gln345, Asn346, Ile347, His363, Val412)
		WITH NAD^+/ NO WATER	-10.03 [c]	Inhibition site (NAD, Ala262, Ile270, Phe273, Ile279, Phe297, Ile316, Gln345, Asn346, Ile347, Asp348)
			-8.01 [c]	Binding site (NAD, His363, Val412, Phe413, Phe414, Gly415, Glu416, Lys444, Val445, Arg446)
SIRT2	Blind	NO NAD^+/ NO WATER	-9.91	Inhibition site (Ile93, Pro94, Phe96, Phe131, Leu134, Ala135, leu138, Tyr139, Pro140, Ile 169, Phe190)
		WITH NAD^+/ NO WATER	-10.03	Inhibition site (NAD, Ile93, Pro94, Phe96, Leu134, Ala135, Leu138, Tyr139, Pro140, Ile 169, Phe190)
	Focused	NO NAD^+/ NO WATER	-10.15	Inhibition site (Ile93, Pro94, Phe96, Phe131, Leu134, Ala135, Leu138, Tyr139, Pro140, Ile 169, Phe190)
		WITH NAD^+/ NO WATER	-9.81	Inhibition site (NAD, Phe96, Leu103, Phe119, Phe131, Leu134, Ile169, His187, Phe234)

[a] Two clusters of conformations under blind docking simulation are reported because they have similar energies and different interaction region on the receptor.

[b] Three clusters of conformations under blind docking simulation with rigid NAD^+ are reported because they have different interaction region on the receptor.

[c] Two clusters of conformations under focused docking simulation with rigid NAD^+ are reported because they have different interaction region on the receptor and different number of poses in cluster (10 vs 33).

[d] Residues interacting with cambinol are reported in parenthesis; residues forming H-bonds are underlined.
